# Nanomaterials: an overview of current trends and future prospects in neurological disorder treatment

**DOI:** 10.1186/s12967-025-06877-6

**Published:** 2025-12-01

**Authors:** Deboral Eshak, Mohanapriya Arumugam

**Affiliations:** Department of Biotechnology, School of Bioscience and Technology, Vellore Institute of Technology, Vellore, Tamil Nadu 632014 India

**Keywords:** Nanomaterials, Blood–brain barrier, Therapeutics, Neurological ailments, Metal oxide nanoparticles

## Abstract

The World Health Organization (WHO) has identified neurological disorders (NDs) as one of the major health concerns worldwide, resulting in high mortality rates. NDs are conditions affecting the central and peripheral nervous systems, including the brain, spinal cord, cranial nerves, peripheral nerves, nerve roots, neuromuscular junctions, and muscles. These neurological diseases include Alzheimer’s disease, Parkinson’s disease, glioma/brain cancer, Huntington’s disease, amyotrophic lateral sclerosis, multiple sclerosis, neuroinfections, ischemic stroke, trauma, hypoxia/anoxia, and depression. Unfortunately, these disorders remain difficult to treat due to the limited ability of conventional drugs to cross the blood–brain barrier (BBB) and achieve significant pharmacological effects in the brain. There is an urgent need to develop methods that can enhance drug efficacy and bypass the BBB. The application of various nanomaterials represents a promising approach to address these neurological disorders. Drugs incorporated with nanomaterials help improve therapeutic outcomes, reduce toxicity, provide better stability, enable targeted delivery, and enhance drug loading capacity. Numerous types and morphologies of inorganic and organic nanomaterials are increasingly employed for treating NDs, including quantum dots, dendrimers, metal nanoparticles, polymeric nanoparticles, liposomes, carbon nanotubes, metal oxide nanoparticles, and micelles. Their exceptional properties such as sensitivity, selectivity, and potential to bypass the BBB make them suitable for both diagnosis and treatment of NDs. In this review article, we briefly summarize the etiology and pathophysiology of various NDs along with current literature highlighting the use of nanomaterials for treating neurological disorders.

**Graphical Abstract:**

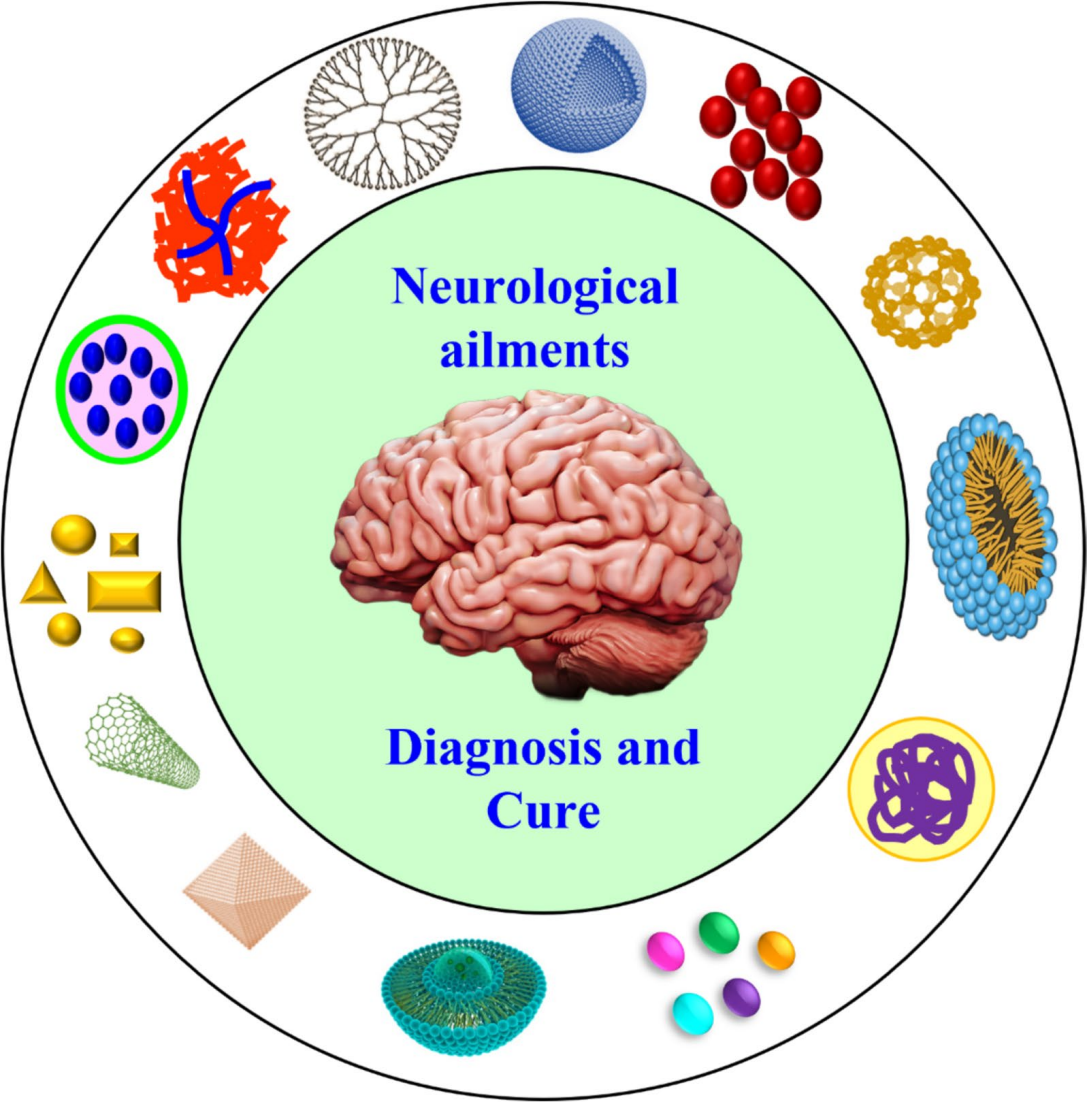

## Introduction

Recently on world stroke day, World Health Organization (WHO) produced a statistical report on neurological ailments stating that stroke stands out in second place for the cause of mortality worldwide, trailed by Alzheimer’s disease and dementias [1]. Earlier report by WHO specified that 68% of mortality in the world is due to brain diseases. Dementia is an ailment connected with harm of memory in aged people which is more frequent in developing nations. It is appraised to rise to 71% in 2040 [2].

Neurological disorders (NDs) are the utmost disturbing and tough ailments of the central nervous system (CNS) and are considered as a foremost menace to communal well-being [3]. They denote the damage of neurons’ structure and functioning. There are dissimilar NDs such as Alzheimer′s, Parkinson′s, Huntington′s, and prion diseases. Causative reason for each disorder is varied. Few NDs causes loss of memory and intellectual impairment, whereas some disturb the capability of the individual to talk, move, and respire [4]. Some other NDs are caused by infection in the brain through bacterial, viral, fungal and bloodsucking mode of transmission. They are Mycobacterial tuberculosis, Neisseria meningitides, Human Immunodeficiency Virus (HIV), Enteroviruses, West Nile Virus, Zika, Cryptococcus, Aspergillus, malaria, Chagas. Symptoms of neurological ailments ensue as a consequence of contamination or because of immune response [5–8]. Central nervous system (CNS) and peripheral nervous system (PNS) diseases represent distinct pathological entities with characteristic features that guide diagnosis and treatment. CNS disorders, affecting the brain and spinal cord, typically present with upper motor neuron signs including hyperreflexia and spasticity, and often involve cognitive impairment when cortical regions are affected [9]. These conditions, including multiple sclerosis, Alzheimer's disease, and stroke, present unique treatment challenges due to the blood–brain barrier limiting medication penetration [10]. In contrast, PNS disorders involve cranial and spinal nerves outside the CNS and manifest with lower motor neuron signs such as hyporeflexia, fasciculations, and muscle atrophy [11]. Peripheral neuropathies, including Guillain–Barre syndrome and diabetic neuropathy, often follow specific nerve distribution patterns and generally have better regenerative potential than CNS diseases [12]. Diagnostic approaches differ significantly, with CNS disorders typically evaluated using MRI and cerebrospinal fluid analysis, while PNS conditions rely heavily on electrophysiological studies and, occasionally, nerve biopsies [13].

The prevalence of neurological disorders (NDs) continues to rise with increasing global population and aging demographics. Diagnosis and treatment of NDs present significant challenges due to the complexity and vulnerability of the central nervous system (CNS). Multiple barriers impede effective therapeutics, including both biological and conceptual obstacles. The blood–brain barrier (BBB), a highly selective semipermeable border, restricts the passage of many therapeutic agents into the CNS, presenting a significant challenge for drug delivery. However, attributing treatment difficulties solely to the BBB oversimplifies the issue. Other critical challenges include the multifactorial nature of most neurological conditions, incomplete understanding of disease mechanisms, progressive neurodegeneration that often precedes diagnosis, limitations in targeting specific neural populations, and the restricted regenerative capacity of neural tissue. While the BBB remains an important consideration in CNS drug development, advances in delivery strategies such as nanoparticles, receptor-mediated transport, and focused ultrasound techniques are increasingly addressing this specific challenge. The development of effective treatments must therefore adopt a multifaceted approach that addresses not only drug delivery across the BBB but also tackles the underlying disease mechanisms, promotes neuroprotection, and potentially enhances neural regeneration. This characteristic feature of BBB is believed to be the foremost task in the growth of medical fraternity. Requirement for progress of therapeutic approaches which can overcome the BBB and likewise can enhance the drug delivery [13–17].

Nano-neuroscience is a new field that combines nanotechnology and neuroscience for effective diagnosis and cure of multiple neurological ailments. This field makes use of development of confined, atomic level nanomaterials with cellular functions to overcome neurological diseases [18]. Nanomedicine offers promising strategies for treating neurological disorders by addressing key challenges like blood–brain barrier penetration and targeted drug delivery. Liposomal nanocarriers have shown efficacy in delivering therapeutic agents to the brain, with clinical success demonstrated in treatments like AmBisome for cryptococcal meningitis [19]. Polymeric nanoparticles, particularly PLGA-based systems, have advanced in preclinical studies for neurodegenerative diseases by encapsulating drugs like levodopa for Parkinson's disease and providing sustained release profiles [20]. Gold nanoparticles functionalized with specific peptides have demonstrated potential for Alzheimer's disease by targeting amyloid plaques and modulating microglial activity in mouse models [21]. However, translational challenges remain significant, including concerns about long-term biocompatibility, consistent manufacturing at clinical scale, and developing disease-specific targeting strategies that function effectively in the complex human CNS environment.

In order to overcome the BBB, nanotechnology is used in recent days with minimal toxicity for the delivery of therapeutic drugs [22]. Nanotechnology emphasizes the utilization of nanomaterials with size 1–100 nm [23, 24]. This technology opens new doors in medicinal field, since many nanoparticles have been used in the studies related to brain disorders, such as gold nanoparticles, metal oxide nanoparticles, micelles, polymeric nanoparticles, quantum dots, silica nanoparticles, etc. [25, 26]. Owing to notable properties of these nanomaterials like smaller size and high surface to volume ratio, they are widely useful for the interaction of blood circulatory system at molecule level with better stability. Also, the use of chemo drugs causes various side effects like stomach allergy, neurotoxicity, lack of appetite. Drug encapsulation using nanomaterials like organic and inorganic nanoparticles helps in disabling the difficulties associated with drug delivery [27]. Over few decades, various categories and morphologies of nanoparticles are extensively utilized to cure diverse forms of NDs [28–30].

Researchers have succeeded in the design of several nanomaterials in the arena of nano-neuroscience for the analysis and cure of various neurological disorders [31]. In this review article, we shortly deliberated the categories of nanomaterials and the development of nanotechnology in the arena of neurosciences. This review article will throw a light on future generations for the development of novel devices from nanomaterials to overcome neurological illnesses.

*Various Neurological ailments and its handling* Neurological ailments are mainly classified as degenerative disorders which includes Multiple Sclerosis (MS), Amyotrophic Lateral Sclerosis (ALS), Alzheimer’s disease (AD), Parkinson’s disease (PD) and Huntington’s disease (HD), tumors including glioma and benign tumors, stroke including ischemic stroke, hemorrhagic stroke and atherosclerosis, Hypoxia/anoxia and infectious NDs.

**Fig. 1:**
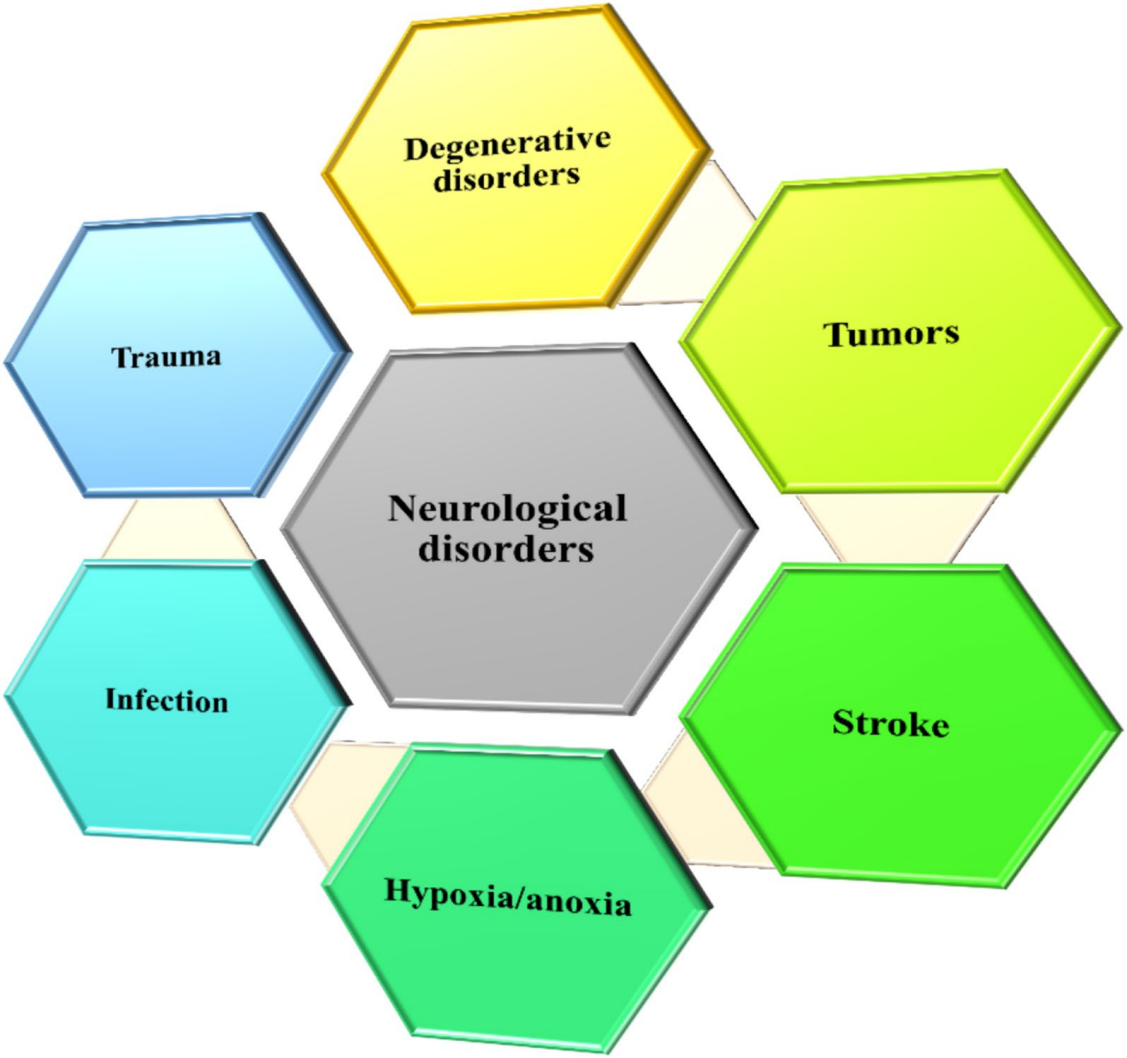
Major types of neurological disorders

Particular reason for neurological ailments varies. Some reasons are genetic mutation, hereditary abnormalities, infections leading to inflammation, lifestyle, malnourishment and injury to backbone and brain [32, 33]. Figure 1 depicts the various neurological disorders.

**Fig. 2:**
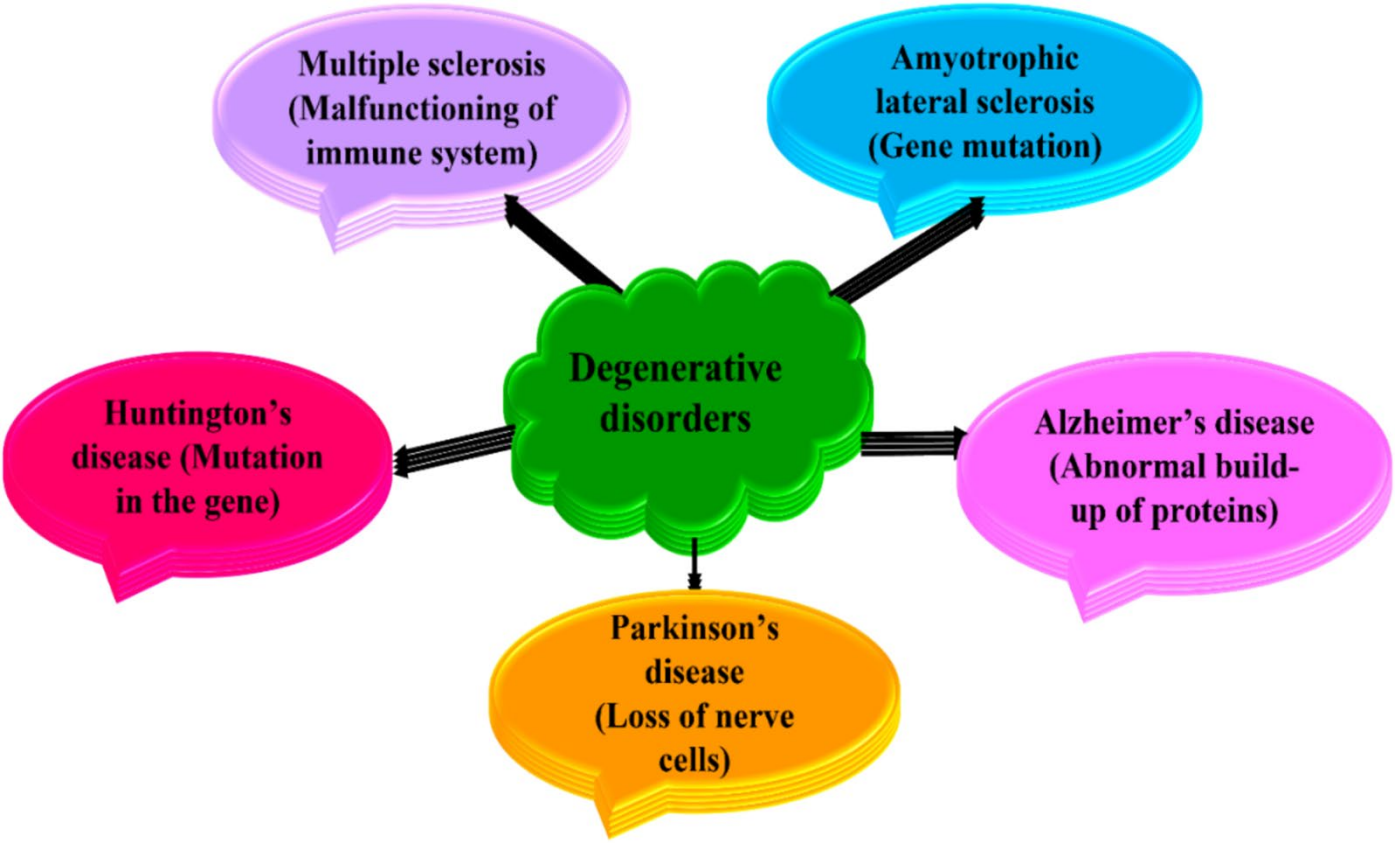
Major causes of neurodegenerative disorders

*Degenerative disorders* Degenerative disorders are due to the advanced damage to the neurons’ structure and function. The progression of such damage is said to be neurodegeneration. This neurodegeneration causes issues in mobility, inflammation to the brain tissues, oxidative stress occurring in brain at varied levels. These disorders are generally incurable ensuing in cell mortality. Degenerative disorders are caused by various factors (Fig. 2) such as malfunctioning of immune system, gene mutation, abnormal build-up of proteins and damage of nerve cells [34, 35].

*Multiple Sclerosis (MS)* Multiple sclerosis (MS) is long-lasting disorder which attacks immune system, damages the protective covering of the CNS, with a reverting/remitting (RR) or an advanced progression which is trailed by destruction to axon leading to paralysis, with symptoms of muscle feebleness, feeble impulses, muscle contraction, trouble in mobility, lack of coordination, imbalance, dizziness, tiredness, and aching [93]. Some other symptoms include damage to nerves of eye, vision loss, damage to pyramidal path, ataxia, tremor, damage to stomach and bladder, malfunctioning of sexuality, hopelessness, nervousness, difficulty in swallowing, loss of memory, trouble in sleep, etc. MS is considered as a multifactorial disorder with an existence of 2–3 times more in women than men. The immune progressions led to the outburst of some therapies. Yet some curative methods are efficient for MS [36].

*Amyotrophic Lateral Sclerosis (ALS)* Amyotrophic lateral sclerosis (ALS) is due to mutation in the gene causing damage to the motor system. Herein, muscle gets tender and waste owing to the injury of superior and inferior motor neurons. Muscle failure extends to other parts of body also. Once the disease start is begun, life is limited to 2–5 years associated with malfunctioning of muscles in the respiratory path. ALS patients are classified into two categories such as sporadic ALS where the disease is not inherited and autosomal ALS where it is inherited from ancestral members. Reason for ALS is not exactly known and its of multiple cause [37]. It is a serious disorder to the brain without any cure till date. Therapeutic drugs utilized to cure ALS are less, still combined medications could afford a better cure for ALS [20].

*Alzheimer’s disease (AD)* Alzheimer’s disease (AD) is a communal type of dementia, which harms nearly 50 million people globally. It is feared that due to the increase in population, this statistic may reach up to 150 million by 2050. AD is a lethal concern pertaining to medical, socio-economical areas. Treatment of AD is restricted due to complications in intranasal route of drug administration. Till now, four treatments are accepted by the Food and Drug Administration (FDA) wherein, the pathway includes developing αβ peptide and neurofibrillary tangle of p-tau protein. Still, these therapies include failure owing to the lack of absorption in the cell membranes of neurons, toxicity to neurons and instability. Hence finding out an effective therapy for AD is the need of the hour. In that, emergence of nanodrugs for treating AD could augment the development of research and its clinical importance [38, 39].

*Parkinson’s disease (PD)* Parkinson’s disease (PD) is the most usual neurodegenerative disorders. It was originally defined by Dr. James Parkinson in 1817 as a “shaking palsy.” It is a long-lasting, developing neurogenerative disorder categorized by motor and nonmotor structures. It affects the movement and muscle control of the patient which ultimately has important medical impact on patients, relatives and care providers. The major symptom of PD is that damage of neurons, which primes to damage to neurons in nondopaminergic regions. It is an indication of resting tremor, bradykinesia and stiffness in muscles. There are other nonmotor indications like disorder in sleep pattern, anxiety and intellectual alterations. All these symptoms start much ahead of motor features. Various health factors and genetic mutations linked with PD like oxidative stress, generation of free radicals and ecological contaminants. Researchers are thriving to find solution for the fortification and prevention of PD [40–42].

*Huntington’s disease (HD)* Huntington’s disease (HD) is due to the CAG expansion in the HD gene. Globally, occurrence of HD is calculated to be 2.7 per 100,000 persons. It is categorized by striatum and cortex neurodegeneration. First and foremost, indication comprises intellectual defects and movement instabilities which increases with time. This disease is an intermittent, congenital, neurodegenerative state which causes advanced motor inability, psychiatric indications, little difficulty in executing actions, depression and intellectual issues. Most of the patients in advance levels lack issue solving and organization ability which leads to unemployment. In final stage, the patients become bedridden, needs feeding tubes, loss of speech, etc. Diagnosis can be done at any time in a person’s life, but in many situations, it happens in middle adulthood. Mortality occurs at 18 years after indications starts, with pneumonia infection. Pre-clinical treatment studies include modulation of autophagy, genetic alterations, use of nanodrugs and stem cell therapy [43, 44].

**Fig. 3:**
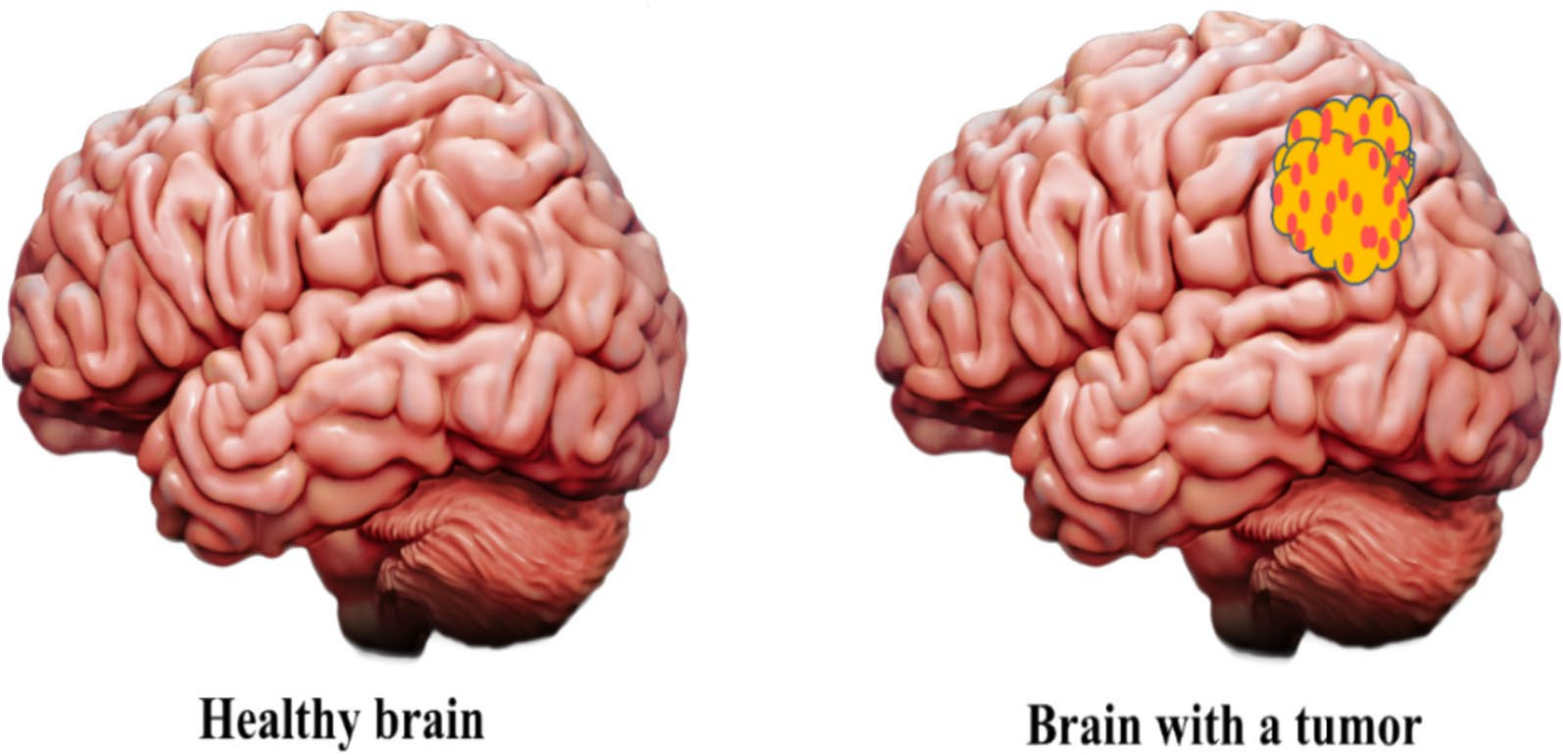
Healthy human brain vs tumors in human brain

*Tumors* Tumors can be of malignant (glioma) which is cancerous and benign which is non-cancerous as shown in Fig. 3. Both are associated with uncontrolled development of cells within brain [45, 46].

*Glioma Brain Cancer* Glioma is the brain cancer which is said to be dangerous cancer instigated in the brain region [94]. It is the 10th foremost reason for mortality among cancer patients across the world. Herein, prognosis and treatment are low among patients. The survivance degree is only 14 months after finding out the disease. Generally, curing glioma involves surgical method, chemo-based treatment, and radio-treatment. But these methods are lethal and cannot fully cure due to the side effects [45, 46].

*Benign tumors* Benign non-cancerous tumors are meningioma and lymphoma, arising around the lymph, brain and backbone [45, 46].

**Fig. 4:**
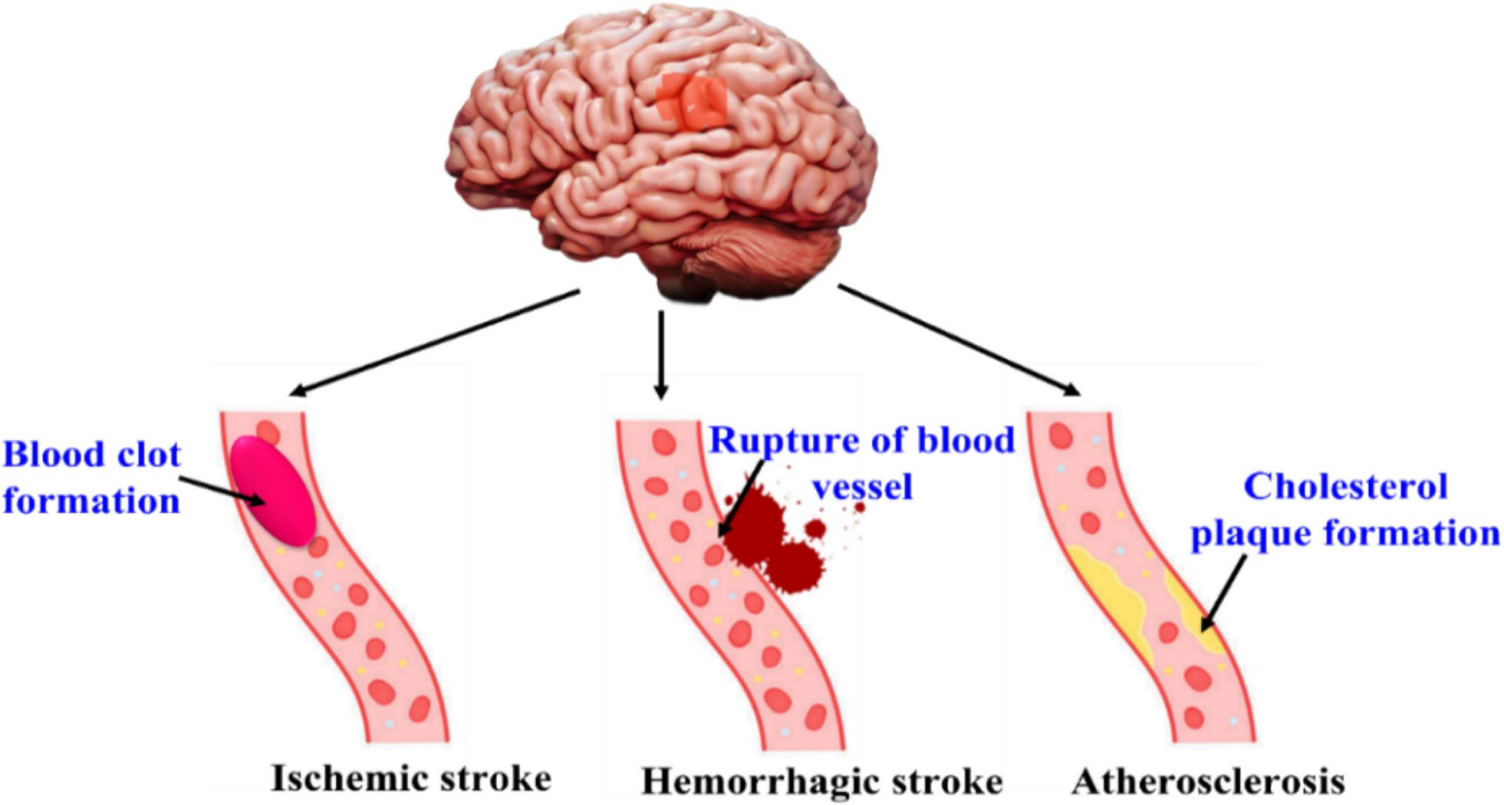
Three types and causes of stroke in human

*Stroke* Stroke is another important reason of mortality universally. Nearly, 13.7 million individuals are affected with stroke and mortality is around 5.5 million people every year. It is a neuro ailment owing to block in blood vessels. These blood clots formed in the brain disrupts the flow of blood, clogging the arteries, leading to breakage of blood vessels with simultaneous bleeding [95]. These causes immediate mortality of cells in the brain mainly due to shortage of oxygen supply. It leads to despair and dementia. Strokes are of three varieties which are ischemic stroke, hemorrhagic stroke and atherosclerosis which are mentioned in Fig. 4. Ischemic stroke is the most prevalent type of stroke wherein blood clot is formed in the blood vessels. Estrangement of blood vessel, bleeding, abnormal accretion of blood in the brain is called as hemorrhagic stroke. Atherosclerosis involves build-up of cholesterol plaque in the arteries resulting in decreased blood circulation to brain parts [47–50]. Hypertension, diabetes mellitus, cardiac infarction, smoking, hyperlipidemia, drug abuse, inflammation and obesity are the risk factors associated with stroke. There are novel ways in developing new drugs by focusing on brain endothelium and provocative neutrophils to enhance the present treatments for stroke [51].

**Fig. 5:**
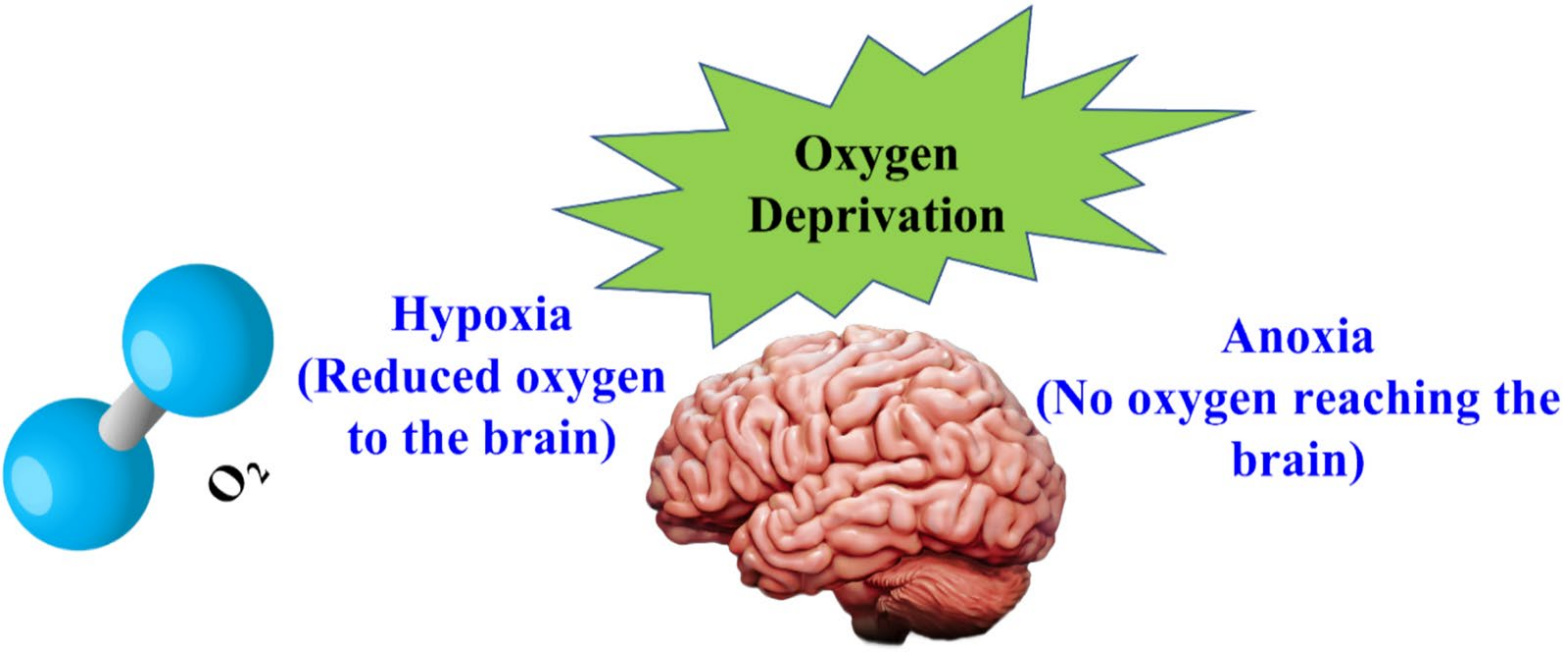
Causes of Hypoxia/anoxia in human brain

*Hypoxia/anoxia* Hypoxia is a condition where oxygen supply is reduced to short period. Acute hypoxia is caused in ischemia and chronic hypoxia is caused in renal disorder and cancer. Anoxia is a condition where no oxygen reaches the brain [96]. Both hypoxia and anoxia are linked with lack of sufficient airway immediately as depicted in Fig. 5, utilizing adequate oxygen to saturate blood, providing support to the cardiac system when required and treatment of pneumonia for respiratory issues [51, 52].

*Infection* Infection in the brain and its tissues are predominantly associated with infections originated from the existence of lethal bacteria, virus, fungi and parasites. These pathogens cause inflammation in the brain resulting in neurological diseases which are life threatening and needs proper treatment. It also demands suitable supervision in case of therapeutic emergencies [7]. Symptoms of such infections results in sudden occurrence of high temperature, pain and tightness in neckline. Contagion in the CNS results in encephalitis, that includes fever, damage to neurofunctions and seizures. In general, this CNS infection is within small region of focal lesions that are common in immunosuppressed people [7, 48].

*Bacterial infection* CNS infection is caused by numerous bacteria. It differs from focal infections, like abscesses, meningoencephalitis. Causative situations include spread from different sites, surgical or trauma-based infections. Bacterial meningitis differs from different age group and based on resistance. Common bacterial infection arises from *Streptococcus agalactiae, Escherichia coli, Streptococcus pneumonia* and *Neisseria meningitidis.* Among them, *Streptococcus pneumonia* and *Neisseria meningitidis* predominant in causing bacterial meningitidis. Vaccination with pneumococcal and meningococcal conjugates has considerably decreased the problem of bacterial meningitis [7].

*Viral infection* Viral infection is usually caused by Enteroviruses and Coxsackievirus B. These 2 viruses are often found in patients with aseptic meningitis. Enterovirus resides in the upper portion of respiratory tract and in intestinal epithelial cells. It enters blood circulation path and CNS via infested immune cells. Enterovirus meningitis is benign, self-liming state. Other viruses responsible for viral meningitis are Herpes simplex virus 2, varicella-zoster virus, Epstein-Barr virus. Mumps virus causes parotitis and loss of hearing, which occurs mostly in unvaccinated populations. Production of novel immunomodulatory and immunosuppressive medications will expand the cure for such viral infections [7].

*Fungal infection* Dissimilar to bacteria, fungi are saprophytic. Fungal infections in CNS are unscrupulous, ensuing from hematogenous distribution in immunocompromised masses. These infections arise from fungal spores during trauma or surgery. Shape of the fungus plays vital role in pathogenesis of CNS lesions. Fungi which form as budding yeast outside causes meningitis (dimorphic fungi) or meningoencephalitis (*Cryptococcus* and *Candida* species). Most invasive type are those show yeast-to-hyphae conversion (e.g., *Candida albicans*) which causes brain abscesses and mortification. Large hyphae (filamentous fungi) have macrovascular invasion, which causes hemorrhagic stroke, aneurysms, and cerebral abscesses. The most common nosocomial infection is candidiasis and the most frequent fungal infection is cryptococcal meningoencephalitis [7, 53, 54].

**Fig. 6:**
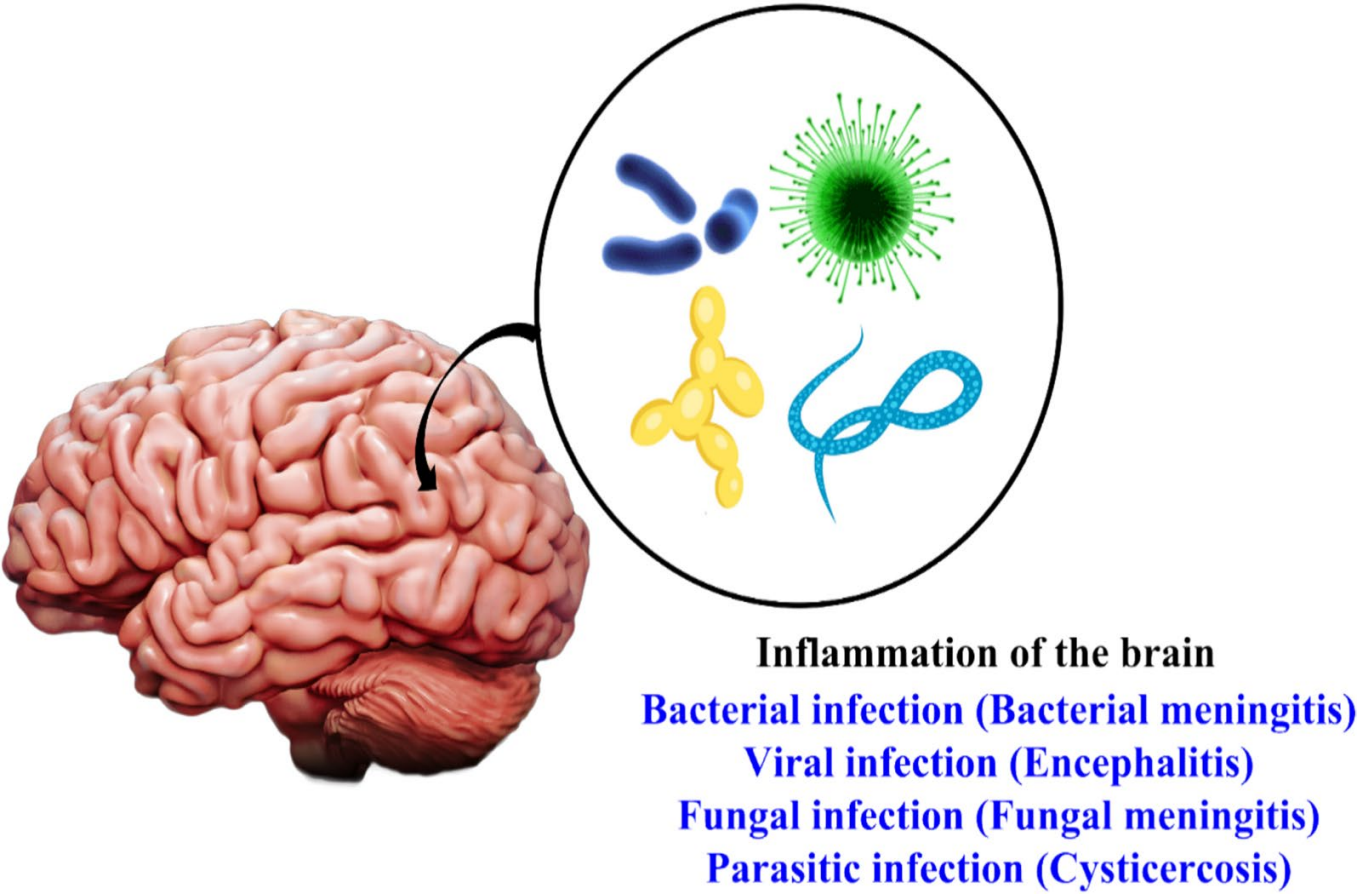
Various types of infections in human brain

*Parasitic infection* Parasitic infection is further common in low and middle earning nations. Unicellular and multicellular organisms like protozoa and worms causes parasitic infection. Life threatening meningoencephalitis (sleeping sickness) is caused by *Trypanosoma brucei.* Neurocysticercosis is instigated by larval cysts of the tapeworm *Taenia solium* which results in increased illness and death. Novel curative method targeting such CNS infections is important need in enhancing the well-being of people across the world as involved in Fig. 6 [7].

**Fig. 7:**
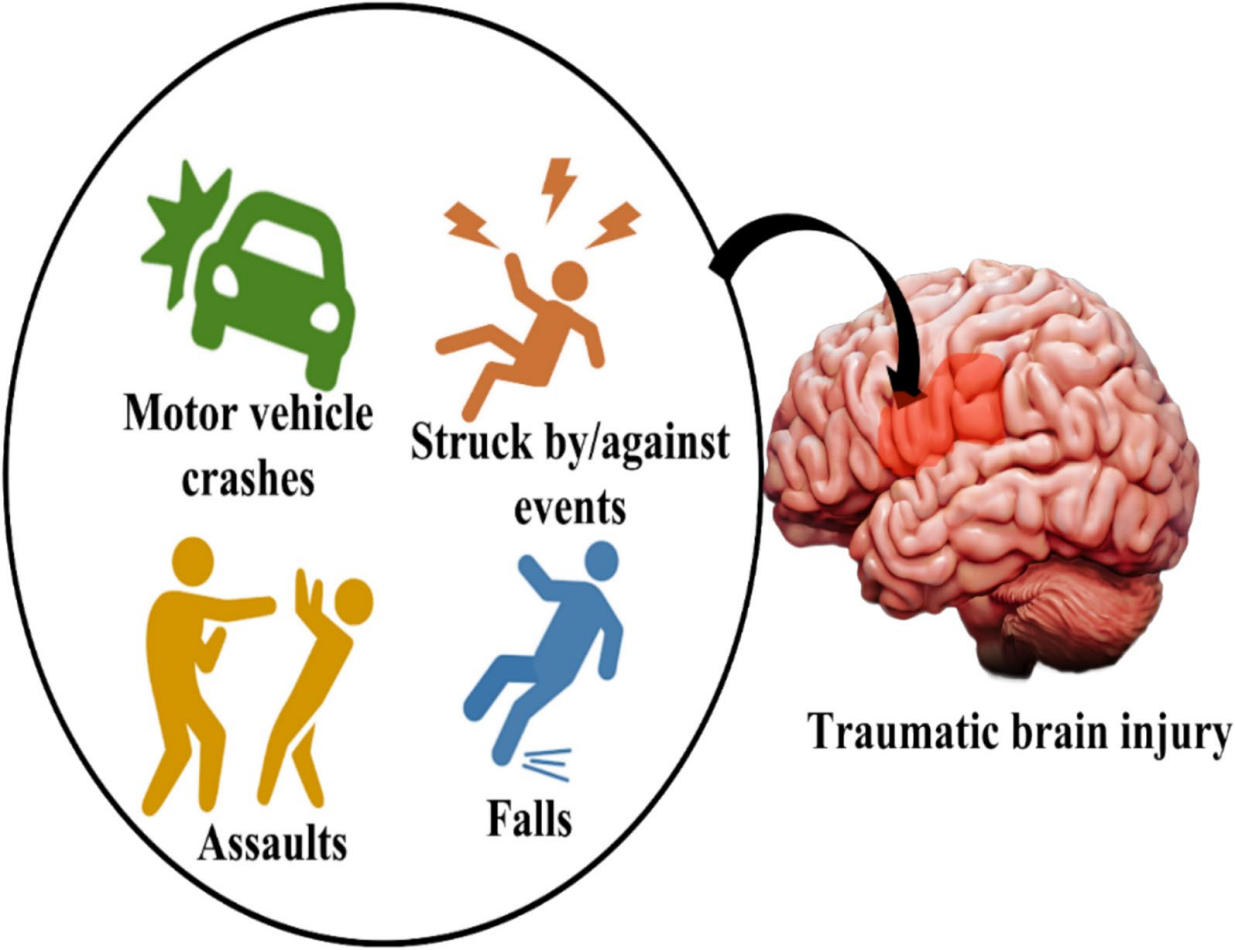
Trauma due to injuries in brain

*Trauma* Traumatic brain injury (TBI) is one among main reason for demise, impairment and morbidity among people of all age. Worldwide, 50 million people are affected by TBI annually. TBI is mainly caused due to motor vehicle crashes, struck by/against events, assaults and fall as shown in Fig. 7. TBI causes stress to brain muscles resulting in an impairment, excitotoxicity, damage to mitochondria, swelling, apoptosis, and development of fluid in brain tissue [97]. All these leads to extended coma, headache, blood leakage, vomiting sensation, aphasia, amnesia and change in behavior like extreme nervousness and anger soon after TBI. Treatment for TBI includes diuretic medication, rest and craniectomy [55, 56].

**Fig. 8:**
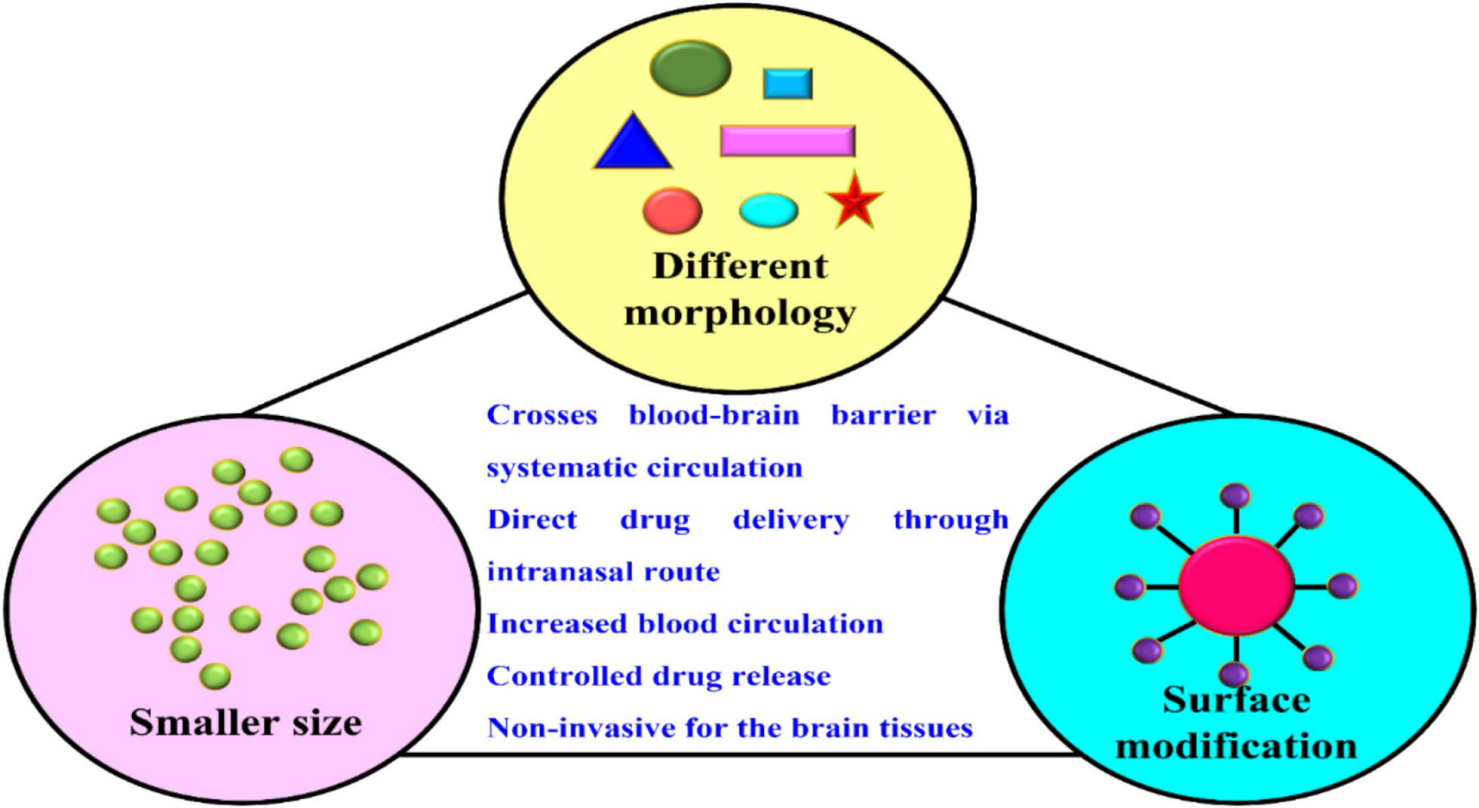
Therapeutic properties of nanomaterials in the cure of NDs

*Properties of nanomaterials for targeted drug delivery to cure neurological disorders* Varied morphologies [57, 58] size, surface to volume ratio, charge, space confinement, surface topography, solubility, magneto-optical properties [59] and dispersion capacity of nanomaterials makes it appropriate for multiple biomedical usages [60]. For the NDs, therapeutic advantages of nanomaterials such as blood–brain barrier crossing ability through efficient circulation in the blood, direct mode of drug delivery onto the site via intranasal path, enhanced blood flow, control of drug release with less toxicity enhances the chances of administrating various nanomaterials in the treatment of NDs as depicted in Fig. 8 [98]. Moreover, research findings states that nanomaterials are non-invasive for the brain tissues which is more advantageous in the field of nanoneuroscience [61]. In particular, magnetic nanoparticles, especially Fe_3_O_4_ has excellent applications in targeted drug delivery due to the superparamagnetic nature [62]. Smaller sized nanomaterials with higher surface area owns better drug dissolution in blood circulation system and minute tissues in brain. These nano level materials coordinate well into biological system hence finding its applications in biomedical devices [63]. Surface modification with particular ligands facilitates the biocompatibility and reduces the toxicity. For example, integration of human albumin on the surface of NPs reduced the toxicity and provided better targeted activity with superior cellular internalization [64].

**Fig. 9:**
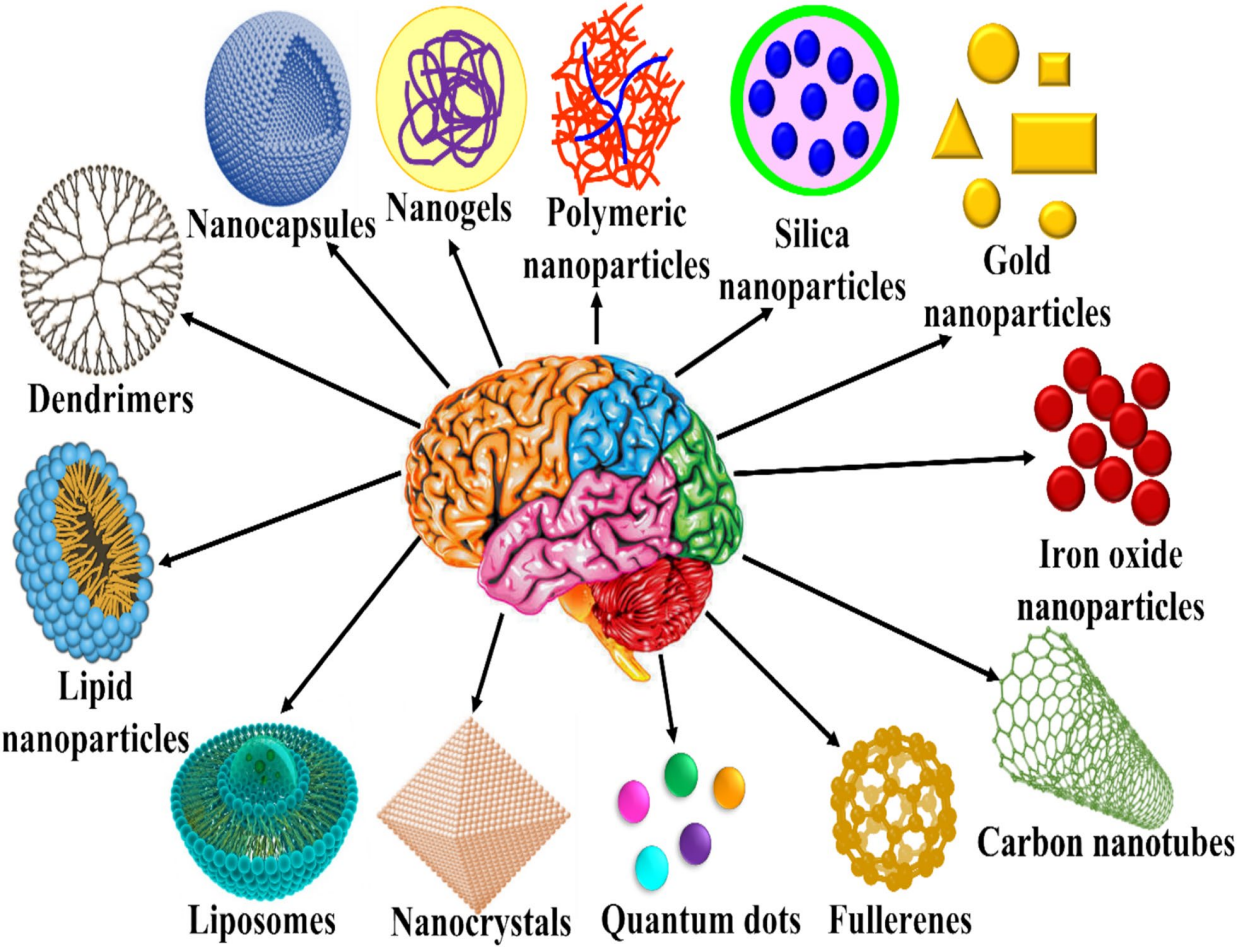
Various nanomaterials to cure neurological disorders

*Categories of Nanomaterials for neurological disorders* Nanomaterials can be broadly classified into three categories such as organic nanomaterials (liposomes, lipids, polymeric micelles, dendrimers), inorganic nanomaterials (gold nanoparticles, silver nanoparticles, silica nanoparticles, carbon nanotubes, quantum dots) and hybrid nanomaterials (e.g., nanogel, graphene oxide) as provided in Fig. 9 [65]. These nanomaterials find applications in the treatment of various neurological ailments like Multiple Sclerosis (MS), Amyotrophic Lateral Sclerosis (ALS), Alzheimer’s disease (AD), Parkinson’s disease (PD) and Huntington’s disease (HD), tumors including glioma and benign tumors, stroke including ischemic stroke, hemorrhagic stroke and atherosclerosis, Hypoxia/anoxia and infectious NDs [98].

*Organic nanomaterials* Organic nanomaterials with natural polymer coatings are within the range of 10 to 1000 nm, which are cost effective. These nanomaterials are environmentally friendly and biodegradable when administered in biological systems [99]. Most used artificial polymers are polylactic acid, polyglycolic acid, polylactide-co-polyglycolic acid and polymethyl methacrylate, while natural polymers are alginate, chitosan, albumin and gelatin [66]. Pharmacokinetics is determined by the polymer structure and insnaring method. For the cure of Alzheimer and Parkinson disease, these organic nanomaterials are widely used owing to their capability to cross BBB and targeting on mutagenic proteins. Also, these nanomaterials are highly stable and can be utilized to neglect macrophages of the reticuloendothelial system. Often these nanomaterials are used as carriers of medications to the central nervous system. Additional benefit of this classification is that it assures elimination of nanomaterials by the outflow structures and consent of drug to brain. Caution should be there in elimination of organic solvents during synthesis for the safe administration of such nanomaterials into CNS. Nano capsules and nanospheres are the forms of organic nanomaterials.

*Inorganic nanomaterials* Inorganic NPs, in specific metal, semiconductor, and metal oxide NPs, are categorized by sole intrinsic optical, electrical, and magnetic properties which gains attention among the Scientists, especially for medicinal applications [100]. By modifying precise constraints like size, morphology, configuration, assembly, and absorbency, it is conceivable to enhance the biotic usage and functionalize its exterior layer by means of ligands and polymers. Silver NPs (AgNPs), iron oxide NPs (IONPs), and titanium dioxide NPs (TiO_2_ NPs) are primarily pragmatic in bioimaging for disease finding [67]. Though, numerous inorganic NPs, like gold NPs (AuNPs) and silica NPs (SiO_2_ NPs), are applied as nanocarriers to influence the CNS. Furthermore, inorganic NPs are considered by long duration improved penetrability and the retaining consequence, that aids them in cure of brain tumor [68]. Use of these NPs for application in the field of medicine are that they are harmless, hydrophilic, biocompatible, and extremely steady under biological circumstances [69]. Metal NPs, such as AuNPs and AgNPs, portray unusual intrinsic optical properties while IONPs are branded by exclusive magnetic nature. SiO_2_ NPs and TiO_2_ NPs are inorganic ceramic NPs and assures advanced thermal and chemical constancy compared to polymeric NPs. IONPs, and Super Paramagnetic Iron Oxide NPs (SPIONs), can perform as capable theranostic drug carriers. With this aspect, by means of magnetic resonance imaging (MRI), the inorganic core becomes measurable, helping as a contrast agent [70].

*Hybrid nanomaterials* Hybrid nanomaterials have notable advantages in nanoneuroscience owing to their multiple properties. There are different hybrid nanostructures like core shell, yolk shell, heterodimer, Janus shape, nanorod, nano branches have been synthesized widely for biomedical applications. In which, diverse functional groups have been utilized for surface modifications. Owing to the morphology, the surface properties has changed [68]. Notably, hybrid NPs made of gold and iron has exhibited better activities against cancer cells and served as a suitable agent for drug delivery system. Apart from these, multimodal imaging, therapy, biosensing, theragnostic properties were displayed by these hybrid nanomaterials which are shown in Table 1 [71]. Some hybrid NPs such as polymeric NPs, solid lipid NPs, liposomal nanoparticulates, nanomicelles and dendrimers with inorganic structures in the form of carbon dots, carbon nano-onions, etc., offer a huge range of structural behaviors favorable for active drug delivery to the CNS [70]. Use of lipid/polymeric hybrid NPs facilitated the cure of degenerative illnesses like Alzheimer and Parkinson diseases. Herein, transfer of drugs transversely to the BBB and improvement of pharmacokinetic outline is effective by means of hybrid NPs. Use of such formulations enables the value of therapeutics and drug transfer to appropriate portion of the brain for regeneration [72]. Biopolymeric NPs such as proteins-polysaccharides, exhibits augmented glioma cure by specific target over the site. Surface modifications with peptides provided improved cure for brain tumor [73]. Mixtures of magnetic/luminescent, plasmonic/catalytic, magnetic/catalytic nanomaterials widely enhanced the biological applications such as biomarkers, imaging, diagnosis tools, targeted drug delivery to particular site. All these properties lead to new area of research in nanoneuroscience as indicated in Table 2 [74].

**Table 1:** Comparative analysis of various nanomaterials for treatment of multiple neuro-disorders with its mode of action

Name of NPs	Properties of NPs	Drug loaded on NPs	Neuro disorder	Model	Mode of action	Ref
Au NPs		–	Alzheimer’s disease	In vitro* activity against* SH-SY5Y cells and in vivo* study using mice model*	Chiral moiety-enabled therapeutic properties	[75]
Ag NPs	Spherical	–	Brain tumour	*In Ovo* study using	Biological activity of U-87 cells	[76]
Polymeric PLGA + PVA NPs	Size = 173.1–500.6 nm	Levodopa	Parkinson’s disease	RSM model	Drug loading efficiency 62.19%	[77]
Clodronate conjugated Liposomes		–	Encephalitis		Reduction in seizures due to depletion in infiltrating macrophages and monocytes	[78]
Ag NPs	Size = 53 nm	Curcumin	Granulomatous amoebic encephalitis	In vitro activity	Amoebicidal activity against *Balamuthia mandrillaris* and *Naegleria fowleri*	[79]
Silica NPs	–	Leptin and Pioglitazone	Amyotrophic Lateral Sclerosis	–	Therapeutic Cocktail Based on Leptin and Pioglitazone	[27]

**Table 2:** Classification of neurological disorders by treatment outcomes

Category	Definition	CNS examples	PNS examples	Key treatments	References
Curable	Complete resolution possible with appropriate intervention	• Bacterial meningitis• Brain abscess • Benign brain tumors • Specific cases of hydrocephalus	• Carpal tunnel syndrome • Compression mononeuropathies • Vitamin B12 deficiency neuropathy	• Antimicrobial therapy • Surgical intervention • Nutritional supplementation	[80, 81]
Manageable	Cannot be cured but can be controlled with ongoing treatment	• Epilepsy • Multiple sclerosis • Parkinson's disease • Migraine	• Myasthenia gravis • CIDP • Diabetic neuropathy • Trigeminal neuralgia	• Disease-modifying therapies • Symptomatic management • Immunomodulation • Neuromodulation	[82, 83]
Currently Incurable	Lack treatments to stop or reverse disease progression	• Alzheimer's disease • ALS • Huntington's disease • Prion diseases • Complete spinal cord injury	• Advanced hereditary neuropathies • End-stage peripheral nerve injuries • Some genetic neuromuscular disorders	• Supportive care • Palliative approaches • Experimental therapies • Rehabilitative strategies	[84–86]

## Tasks and upcoming standpoints

It is reported that use of nanomaterials in neurological ailments do possess some challenges. This leads to the tasks which need to be addressed for effective improvement of drug delivery to brain tissues. Some of the tasks are listed as follows: Nanomaterial’s ability to cross the BBB, maintenance of therapeutic level inside brain, non-degradability of drug loaded NPs, need for low concentration activity, toxicity level of nano coated drugs, sudden immune responses, tedious for the modification of organ in CNS to cope with the medication for neurological disorders [87].

Although, above tasks are existing, nanomaterials-based neuro treatment offers many advantages like lack of phagocytosis and resistance mechanisms, easy admittance to CNS. For the development of nano based drug, it requires a long-term work starting from synthesis, characterization, in vitro and in vivo analysis for clinical level entry. Nowadays, advent of Bioinformatics for the screening of positive hits reduces the time-consuming and affluent wet laboratory processes. It is crucial to screen any formulation via Bioinformatics before cell line studies and animal trials for suitable NPs design for neurological treatments [88].

Major limitation in the treatment of brain disorders is that some drugs cause extreme toxicity while it is spread in CNS. Toxicity arising from NPs predominantly relies on multiple factors such as shape, size, charge, surface area, dispersibility, concentration, path and time of administration, still most of the times toxicity is encountered as result of oxidative stress owing to the generation of free radicals. Such reports were witnessed with the use of Mn [89] and Cu NPs [90]. In addition, ZnO NPs induced apoptosis in stem cells of brain [91]. The long-term use of Ag NPs leads to toxicity in kidney and liver, also in brain while oral route of administration [92]. Hematite NPs while administered through intranasal path, caused neuronal fatty degeneration in hippocampus. These are some limitations with the use of NPs in the cure of NDs. Bearing in mind of above toxicities, it is recommended that utilization of biocompatible and decomposable polymers during the synthesis of NPs lessens the adverse effects and averts the deprivation of NPs, enabling an effective drug release. Apart from the adverse possessions, these NPs possess specific surface charge and some physical properties, makes them suitable candidate in the field of diagnostics. Nevertheless, in future it is highly recommended to do more studies to evaluate and lay down the strategies for overcoming the toxic effects.

## Conclusion

Nearly, 276 million infirmities and 9 million deaths are instigated by NDs. It is alarming that these cases are anticipated to upsurge enormously in near future. Owing to the BBB, it is very tough to cure and handle cases with NDs. Often, entry of drug to targeted site is an issue. This can be solved with the use of NPs based drugs which effectively reaches the targeted site due to their multipurpose properties. Nowadays, nanoneuroscience is attracted by scientists from diverse fraternity of biological science, particularly neurosciences. Unique properties like surface-to-surface ratio, smaller size, varied morphologies, selectivity, sensitivity, charges and steadiness, these nanomaterials offer a better platform in the cure of NDs. Not only the cure, but also the diagnosis of NDs is possible through nanoneuroscience. Pertaining to the cure of NDs, there is still need for the study of pharmacokinetics and pharmacodynamics. For the large scaling up of such technologies needs the pioneering ideas from chemists, engineers, and other researchers. This review paper highpoints the significance of various nanomaterials in developing a curative cause for NDs.

## Limitations

Limitations A significant number of the data referred in this study were based on studies conducted in China and in a few other areas. The limitation on geography might restrict the generalization of these results to other ethnicities and regional populations. Such validation studies in diverse populations should be performed in future research.

## Data Availability

Not applicable.
